# The analysis of virulence factors and antibiotic resistance between *Helicobacter pylori* strains isolated from gastric antrum and body

**DOI:** 10.1186/s12876-019-1062-5

**Published:** 2019-08-07

**Authors:** Ji Won Seo, Jae Yong Park, Tae-Seop Shin, Jae Gyu Kim

**Affiliations:** 1 Department of Biomedical Science, Chung-Ang University College of Medicine, Seoul, South Korea; 2 Department of Internal Medicine, Chung-Ang University Hospital, Seoul, South Korea; 3 Research Institute, Chung-Ang University, Seoul, South Korea; 4 Department of Internal Medicine, Chung-Ang University College of Medicine, 102 Heukseok-ro, Dongjak-gu, Seoul, 06973 Republic of Korea

**Keywords:** Random amplified polymorphic DNA, *Helicobacter pylori*, Virulence factor, Antibiotic resistance

## Abstract

**Background:**

Individuals can be infected with multiple strains of *Helicobacter pylori*. However, the differences among co-infecting strains have not been well analyzed yet. This study aimed to investigate whether the virulence factors and antibiotic resistance patterns of *H. pylori* differ between strains isolated from different locations of the stomach in the same patient.

**Methods:**

*H. pylori* isolates were obtained from the antrum and body of the stomach. Genetic differences were examined by random amplified polymorphic DNA (RAPD) fingerprinting. Antibiotic resistance was assessed using the agar dilution method. Virulence factors were identified by polymerase chain reaction and DNA sequencing.

**Results:**

Among 80 patients, co-infection by two *H. pylori* strains was detected in 10 patients. Among the 10 pairs of *H. pylori* strains, differences in antibiotic resistance patterns were detected in 7 pairs (clarithromycin, 1 patient; quinolone, 3 patients; metronidazole, 4 patients) and differences in virulence factors were detected in 5 pairs. The *cagA* virulence gene was detected in all 10 patients, and 2 patients had *H. pylori* strains with different EPIYA motifs. Differences in *vacA* genotypes were detected in 4 patients.

**Conclusions:**

Co-infection by two *H. pylori* strains was confirmed by RAPD fingerprinting. Frequently, two *H. pylori* strains obtained from a single host differed in their virulence factors and antibiotic resistance patterns. Co-infection by multiple *H. pylori* strains could undermine the success of eradication therapy and should be considered when interpreting the results of antimicrobial susceptibility tests.

**Electronic supplementary material:**

The online version of this article (10.1186/s12876-019-1062-5) contains supplementary material, which is available to authorized users.

## Background

*Helicobacter pylori* is a spiral microaerophilic gram-negative bacterium that colonizes the human gastric mucosa. *H. pylori* is a major cause of gastric and duodenal diseases, and it has been estimated to currently infect half of the world’s population [1, 2]. It is already known that many virulence factors of *H. pylori* are related to the occurrence of various gastrointestinal disorders [3, 4]. The effect of *H. pylori* eradication therapy on gastrointestinal disorders has been vigorously studied. There is evidence that *H. pylori* eradication can cure gastritis, delay or prevent progression to long-term complications, or prevent the recurrence of disease. Even some extragastric diseases are thought to be related to *H. pylori* infection and could potentially be cured with eradication therapy.

Despite these benefits of *H. pylori* eradication therapy, an increasing resistance of the organism to antibiotics is becoming a major concern. Since the antibiotic resistance of *H. pylori* is the most important cause of eradication failure in many regions of the world, the role of antimicrobial susceptibility testing is becoming more important, especially in populations from regions with a high antibiotic resistance or after experiencing eradication failure [5, 6]. In addition, co-infection by more than one *H. pylori* strain in a single patient should be considered when antibiotic resistance is suspected [7–10]. Multiple *H. pylori* strains isolated from the same host might differ in certain characteristics such as their virulence factors or antibiotic resistance patterns. Such co-infections could affect the success of eradication therapy, thereby influencing the treatment course and prognosis of patients with *H. pylori* infection. However, the differences among *H. pylori* strains obtained from the same host in terms of their characteristics have not been well investigated yet.

In our study, infection by two *H. pylori* strains in a single patient was identified using random amplified polymorphic DNA (RAPD) fingerprinting, and the differences in virulence factors and antibiotic resistance between the co-infecting strains were investigated.

## Methods

### Patients and bacterial strains

We retrospectively enrolled 80 patients. Enrolled patients were those who visitied Chung-Ang University Yong-san Hospital between January 2005 and December 2009 due to upper gastrointestinal symptoms and underwent esophagogastroduodenoscopy with biopsies from the antrum and body of the stomach, with successful *H. pylori* isolation from both antrum and body. The patients with positive *H. pylori* culture only from one site, either antrum or body, were not eligible for this study. Totally, 80 pairs of *H. pylori* isolates were obtained from the enrolled subjects. The Institutional Review Board of Chung-Ang University Hospital approved this study [IRB number: C2014247(1444)].

### Histopathologic evaluation of biopsy samples from gastric mucosa

Two gastric mucosal tissues, one from the lesser curvature of the antrum and the other from the lesser curvature of the body, were taken for histologic evaluation. These tissue samples were histologically examined using the updated Sydney system: *H. pylori* density, inflammatory activity (neutrophil infiltration), atrophy, and intestinal metaplasia were assessed and scored as 0 (none), 1 (mild), 2 (moderate), and 3 (marked) [11].

### *H. pylori* culture and genomic DNA isolation

*H. pylori* isolates were cultured at 37 °C on Brucella agar plates (Becton Dickinson, Franklin Lakes, NJ, USA) containing 5% defibrinated sheep blood (Hanil Komed, Seongnam, Republic of Korea) under microaerobic conditions (5% O_2_, 10% CO_2_, 85% N_2_) for 3–5 days. The *H. pylori* isolates subcultured less than 3 times were used in all experiments, and subculture was carried out on blood brucella agar plates under the same conditions. Organisms were identified as *H. pylori* by colony morphology, rapid urease test, *H. pylori*-selective media [Oxoid™ SR 147 supplement (Thermo Fisher Scientific, Waltham, MA) and 5% defibrinated sheep blood], and polymerase chain reaction (PCR) to detect *ureA*.

Genomic DNA extraction was performed by harvesting *H. pylori* subcultured for 3–5 days and using a HiYield™ genomic DNA mini kit (Real Biotech Corporation, Taipei, Taiwan). The isolated genomic DNA was stored at − 20 °C until required for PCR amplification.

### PCR for virulence factors and RAPD fingerprinting

PCR was used to detect the *H. pylori*-specific *ureA* gene, CagA EPIYA motif type, *vacA* genotype, *oipA* status, and *cagA*, *dupA*, and *iceA* genes. RAPD fingerprinting was used to analyze *H. pylori* isolated from the stomach to determine whether there were multiple strains co-infecting a single patient [10, 12–14]. All the primer sets used were selected from previously published literature (Additional file 1: Table S1).

The Ex Taq™ DNA polymerase (Takara Bio, Otsu, Shiga, Japan) was used for PCR amplification, which was performed in a volume of 50 μL, containing 5 μL 10× Ex Taq™ buffer including MgCl_2_, 4 μL dNTP mixture (2.5 mM each), 10 pmole of each primer, and 1.25 U Ex Taq™ DNA polymerase, following previously described methods [12, 15–24]. A GeneAmp® PCR system 2700 (Applied Biosystems, Foster City, CA, USA) was used for amplification.

### Sequencing of CagA EPIYA, *oipA,* V domain of 23S rRNA gene, *gyrA* and *gyrB*

PCR and DNA sequencing were used to confirm whether the *cagA* genes of the isolated *H. pylori* strains had the Western or East Asian type of EPIYA motif [4]. The EPIYA-containing region of amplified *cagA* was confirmed by agarose gel electrophoresis and gel extraction was performed. The electrophoretic bands were cut out and PCR products were extracted from the gel using a HiYield™ gel/PCR DNA mini kit (Real Biotech Corporation). The gel extraction products thus obtained were submitted for sequencing (Macrogen Corporation, Seoul, Republic of Korea). Sequences of the EPIYA-containing regions thus obtained were converted to protein sequences using CLC Main Workbench 5 (Qiagen, Hilden, Germany) and aligned. The sequence of the amplification product of the primers targeting the *oipA* gene was aligned through CLC Main Workbench 5 and the presence of the CT repeat motif was confirmed. Additionally, the mutations of 23S rRNA gene and *gyrA/gyrB* sequence were analyzed to clarify the mechanism of antibiotic resistance for clarithromycin and quinolone, respectively.

### Determination of minimum inhibitory concentrations (MICs) of antibiotics

In our study, we selected the internationally recognized agar dilution method, which can accurately measure MICs and is widely used for antibiotic resistance testing (Clinical and Laboratory Standards Institute document M07-A10). The antibiotics used in the MIC tests were clarithromycin (0.0625–16 μg/mL), levofloxacin (0.125–16 μg/mL), amoxicillin (0.125–16 μg/mL), metronidazole (2–128 μg/mL), moxifloxacin (0.125–16 μg/mL), and tetracycline (0.125–16 μg/mL).

*H. pylori* were inoculated on a Mueller–Hinton agar plate (Becton Dickinson) containing each antibiotic and 5% defibrinated sheep blood. After *H. pylori* were cultured at 37 °C in a 10% CO_2_ environment for 5–7 days, the plate was examined for visible growth and the lowest concentration of antibiotics that completely inhibited growth was determined as the MIC point. MIC testing was performed three times for each strain. Only the MIC point obtained using agar dilution method in previous literature was used to avoid confusion over the interpretation of antibiotic resistance results determined using other MIC methods [25]. Differences of MIC for an antibiotic between two strains cultured from a single patient were considered significant when the MICs differed by 4 times or more. When there was a difference in the presence of resistance even for one antibiotic between two strains cultured from one patient, the strains were defined as having different antibiotic resistance patterns.

## Results

### RAPD fingerprinting and clinical information

**Fig. 1:**
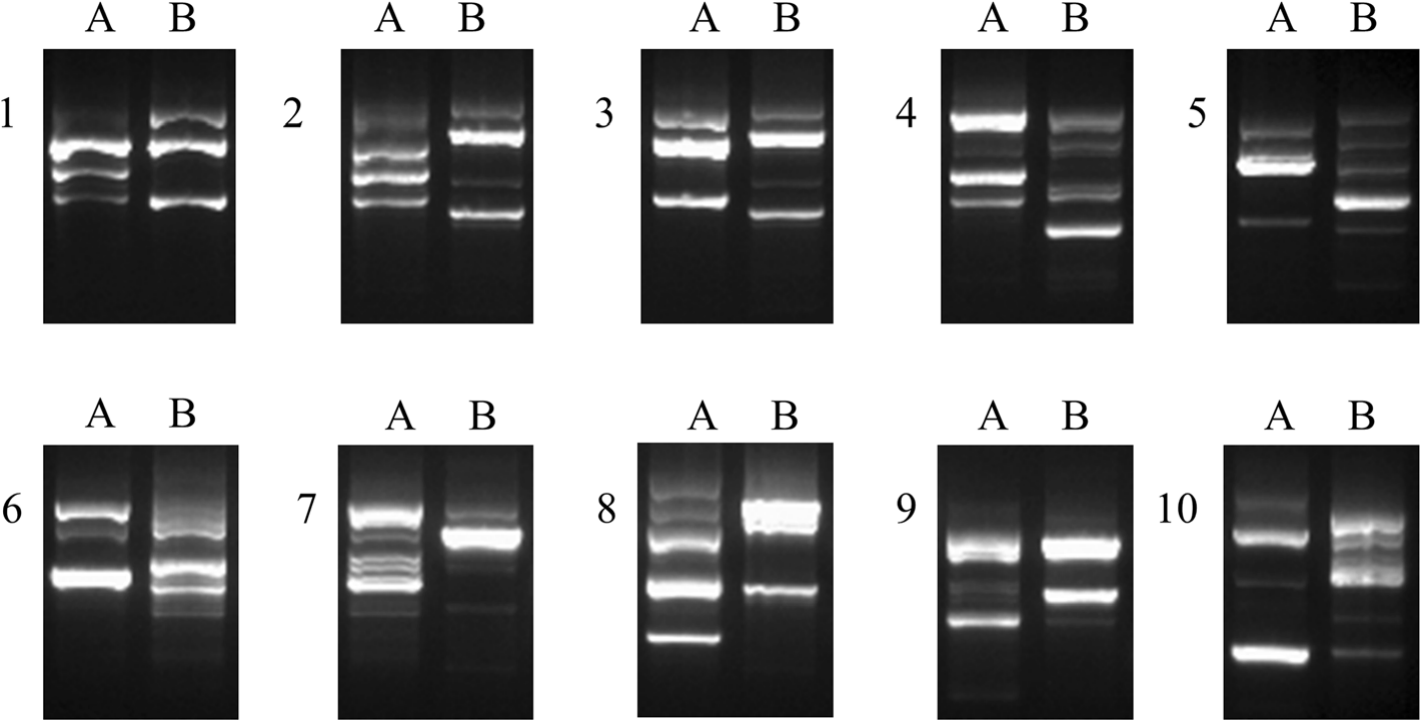
RAPD fingerprinting patterns of *H. pylori* isolates from patients obtained by RAPD analysis with primer 1281. Numbers 1–10 indicate individual patients; A, stomach antrum; B, stomach body

Among 80 patients with successful *H. pylori* isolation from the antrum and body of the stomach, the co-existence of different *H. pylori* strains was detected in 10 patients by RAPD fingerprinting of *H. pylori* (Fig. 1). Furthermore, PCR for the *ureA* gene was performed to confirm the *H. pylori* isolates from 10 patients (6 females, 4 males). The median age of the 10 patients was 57 years (range 40–69). All of the 10 patients were diagnosed with gastritis, and none had gastric dysplasia, gastric cancer or peptic ulcer. They all showed chronic atrophic gastritis, and only one of them had intestinal metaplasia. The degree of atrophy was mild in most of the cases. Detailed clinical information of these 10 patients is shown in Table 1.

**Table 1:** Clinical information of 10 patients

Patient	Pathology			
	Inflammatoryactivity	*H. pylori*	Intestinal metaplasia	Atrophy
1A	1	2	0	1
1B	1	2		1
2A	1	1	0	1
2B	0	1		1
3A	1	2	0	1
3B	1	2		1
4A	1	2	0	1
4B	0	1		1
5A	1	1	0	1
5B	0	1		1
6A	1	2	0	1
6B	0	2		1
7A	2	2	0	2
7B	2	2		1
8A	1	1	0	1
8B	0	1		1
9A	1	1	0	1
9B	0	1		1
10A	1	1	1	1
10B	1	2		1

Numbers 1–10 indicate individual patients; A, stomach antrum; B, stomach body

### Differences in virulence factors

The positivity and subtypes of several virulence factors are described in Table 2. Overall, 5 of the 10 patients (50%) showed differences in the profiles of virulence factors between their two co-infecting *H. pylori* strains. All the *H. pylori* strains obtained from the 10 patients had an s1-type *vacA* s-region. In 4 patients (40%), the co-infecting *H. pylori* strains differed in their *vacA* s1 subtypes. Regarding the *vacA* m-region and i-region, co-infecting *H. pylori* strains with different patterns were isolated in 2 of the 10 patients (20%) (Additional file 2: Figure S1).

**Table 2:** Positivity of *vacA,* CagA EPIYA*, iceA, oipA,* and *dupA* of *H. pylori* in 10 patients

Strain no.	*vacA*			*iceA*	CagA EPIYA	*oipA*	*dupA*
	s-region	m-region	i-region				
1A	s1c	m1	i1	iceA1	ABD	3 + 1 on	–
1B	s1a	m1	i1	iceA1	ABD	3 + 1 on	–
2A	s1c	m1	i1	iceA1	ABD	3 + 1 on	–
2B	s1a	m1	i1	iceA1	ABD	3 + 1 on	–
3A	s1a	m1	i1	iceA1	ABD	3 + 1 on	–
3B	s1a	m1	i1	iceA1	ABD	3 + 1 on	–
4A	s1c	m1	i1	iceA1	ABD	2 + 1 + 1 + 1 on	–
4B	s1c	m1	i1	iceA1	ABD	3 + 1 on	–
5A	s1c	m2	i2	iceA1	ABC	3 + 1 on	–
5B	s1a	m1	i1	iceA1	ABD	3 + 1 on	+
6A	s1c	m1	i1	iceA1	ABD	2 + 1 + 3 on	–
6B	s1c	m1	i1	iceA1	ABD	2 + 1 on	–
7A	s1c	m1	i1	iceA1	ABD	3 + 1 on	–
7B	s1c	m1	i1	iceA1	ABD	3 + 1 on	–
8A	s1c	m1	i1	iceA1	ABC	5 + 3 on	+
8B	s1a	m2	i2	iceA1	ABC	5 + 3 on	+
9A	s1c	m2	i1	iceA1	ABD	2 + 1 + 1 + 1 on	–
9B	s1c	m2	i1	iceA1	ABD	2 + 3 + 1 on	–
10A	s1c	m1	i1	iceA1	ABD	2 + 2 + 1 on	+
10B	s1c	m1	i1	iceA1	ABC	3 + 1 on	–

Numbers 1–10 indicate individual patients; A, stomach antrum; B, stomach body; *oipA* status is expressed as CT repeat number and on/off status. The expression of OipA is regulated by the slipped-strand repair mechanism based on the number of CT dinucleotide repeats in the 5′ signal-sequence coding region the oipA gene (“on” = functional; “off” = nonfunctional)

The *cagA* gene was positively detected in all the strains from the 10 patients, and 2 of 10 patients (20%) were found to have *H. pylori* strains with different EPIYA motifs (Additional file 2: Figure S2) and *dupA* statuses (Additional file 2: Figure S3). In all the strains, the *oipA* gene had ‘on’ status (Additional file 2: Table S2) and the iceA1 allele of *iceA* was detected (Additional file 2: Figure S3).

### Antibiotic resistance of *H. pylori* strains

The antibiotic resistance patterns of all the *H. pylori* strains from the 10 patients are described in Table 3. Among the 10 pairs of *H. pylori* strains, 9 pairs (90%) showed significantly different MIC results, and 7 pairs (70%) showed different profiles of antibiotic resistance. *H. pylori* strains with different antibiotic resistance patterns to quinolone, metronidazole, and clarithromycin were detected in 3, 4, and 1 patients (30, 40, and 10%), respectively. (Additional file 3: Table S3)

**Table 3:** Minimum inhibitory concentration of *H. pylori* in 10 patients

Strain no.	Clarithromycin (> 1.0)	Levofloxacin (> 1.0)	Amoxicillin (≥0.5)	Metronidazole (> 8)	Moxifloxacin (> 1.0)	Tetracycline (> 4.0)
ATCC 43504	0.0625	0.25	0.125	128	0.25	0.125
1A	0.0625 (S)	16 (R)	0.125 (S)	8 (S)	8 (R)	0.25 (S)
1B	0.0625 (S)	0.125 (S)	0.125 (S)	4 (S)	0.125 (S)	0.125 (S)
2A	0.0625 (S)	8 (R)	0.125 (S)	16 (R)	8 (R)	0.5 (S)
2B	0.0625 (S)	0.5 (S)	0.125 (S)	128 (R)	0.5 (S)	1 (S)
3A	0.0625 (S)	0.25 (S)	0.125 (S)	64 (R)	0.25 (S)	0.25 (S)
3B	0.0625 (S)	1 (S)	0.125 (S)	128 (R)	1 (S)	1 (S)
4A	0.0625 (S)	0.5 (S)	0.125 (S)	4 (S)	0.5 (S)	0.5 (S)
4B	0.0625 (S)	0.5 (S)	0.125 (S)	4 (S)	0.25 (S)	0.5 (S)
5A	0.0625 (S)	0.25 (S)	0.125 (S)	4 (S)	0.125 (S)	0.5 (S)
5B	0.0625 (S)	0.5 (S)	0.125 (S)	16 (R)	0.25 (S)	1 (S)
6A	0.0625 (S)	0.5 (S)	0.125 (S)	128 (R)	0.25 (S)	0.5 (S)
6B	0.0625 (S)	0.5 (S)	0.125 (S)	4 (S)	0.25 (S)	1 (S)
7A	0.0625 (S)	0.5 (S)	0.125 (S)	4 (S)	0.5 (S)	0.125 (S)
7B	0.0625 (S)	1 (S)	0.125 (S)	2 (S)	1 (S)	1 (S)
8A	16 (R)	8 (R)	0.25 (S)	128 (R)	8 (R)	0.25 (S)
8B	16 (R)	0.25 (S)	0.125 (S)	128 (R)	0.25 (S)	0.125 (S)
9A	0.0625 (S)	0.25 (S)	0.125 (S)	4 (S)	0.25 (S)	0.5 (S)
9B	0.0625 (S)	0.5 (S)	0.125 (S)	128 (R)	0.25 (S)	2 (S)
10A	0.0625 (S)	0.5 (S)	0.125 (S)	16 (R)	0.25 (S)	2 (S)
10B	16 (R)	1 (S)	0.125 (S)	16 (R)	1 (S)	1 (S)

Numbers 1–10 indicate individual patients; A, stomach antrum; B, stomach body; S, susceptible; R, resistant

## Discussion

In our study, co-infecting *H. pylori* strains obtained from two different gastric locations in a single patient showed different RAPD fingerprinting patterns, and co-infections by two strains were detected in 12.5% of all our tested cases. In addition, half of them had *H. pylori* strains with different profiles of virulence factors. These findings show that patients may be affected by multiple different *H. pylori* strains with varying virulence factors. Similarly, the antibiotic resistance patterns of the co-infecting strains differed in the majority of cases. These results suggest that co-infecting *H. pylori* strains frequently have different antibiotic resistance patterns as well as different virulence factors.

Though there have been some reports showing co-infection with multiple *H. pylori* strains in a single patient, the results have not been consistent among studies [7–10]. In a previous study conducted in Japan, it was suggested that infection with single *H. pylori* strain was very common, while infection with multiple strains was rare (detected in 0 of 30 patients) [26]. The difference in the prevalence of infection by multiple strains detected in our study compared with previous studies might be mainly due to regional differences in the overall prevalence of *H. pylori* infection. Most importantly, the prevalence of *H. pylori* infection in South Korea (54.4%, 1998–2011) [27] appears to be higher than that in Japan (39.3%, 1974–1994) [28]. In regions where the prevalence of *H. pylori* infection is high, the chance of multiple infection with different strains is relatively higher than that in regions with low prevalence of *H. pylori* infection [29]. The prevalence of multiple infection was generally lower in developed countries (0–10%) than the prevalence in developing countries (> 20–35%) [8, 9, 30, 31]. It could be inferred that low *H. pylori* infection rate due to improved hygiene, sanitation, and eradication with antibiotic therapy in developed countries contributed to the low rate of multiple infection in a single host. Although being a developed country, Korea is also well-known for the highest incidence rates of gastric cancer in the world with high prevalence of *H. pylori* infection. Recently, the *H. pylori* prevalence has been steadily decreasing especially among the young population in Korea [32]. Considering these factors, the multiple infection rate of 12% in our study seems a feasible value considering the rates in other developed countries. In addition, the differences of study design, such as type of samples and biopsy locations, could also account for the differences in the multiple infection rates among studies from different regional areas.

Our study not only confirmed the occurrence of co-infections by multiple strains of *H. pylori* using RAPD fingerprinting, but also identified differences between the co-infecting strains in their virulence factors and antibiotic resistance patterns. These are important factors that influence the natural course of *H. pylori*-related diseases, as well as the treatment results and prognosis of the infected individuals.

It has been reported that more than 90% of East Asian *H. pylori* strains are *cagA* positive [33], and the East Asian-type CagA can show increased virulence as compared to the Western-type CagA [34]. In our study, all the tested *H. pylori* strains were *cagA*-positive and almost all of them had the East Asian-type *cagA*, which is similar to the results from previous studies [33]. In addition, almost all the *cagA*-positive strains had the s1/m1-type *vacA*, which presents the most cytotoxic profile among various *vacA* subtypes [18, 35]. Our results, like those of the previous study, showed that all the *H. pylori* strains with *cagA* had the s1-type *vacA*. Some researchers suggest that multiple infection could affect the histologic changes of gastric mucosa [31, 36]. Previous study has shown that there are differences in histological changes in the antrum and the body of the stomach between the patients with mixed *H. pylori* infections and those with single strain infection. Mixed infection was associated with significant higher rate of presence of intestinal metaplasia in the antrum [31]. In another study, mixed *H. pylori* infection was significantly frequent in patients with duodenal ulcer than in those with chronic gastritis [36].

Recently, since clarithromycin has become widely used for various indications including respiratory infections or nontuberculous mycobacterial infections, the prevalence of *H. pylori* resistance to clarithromycin has been increasing, resulting in a worldwide reduction of eradication rates [37, 38]. Resistance to other antibiotics has also been increasing, leading to a steady decrease in eradication rates. This problem is especially a concern in local areas with high clarithromycin, quinolone, or metronidazole resistance. Eradication rates with previously efficacious regimens have been decreasing worldwide [5, 6, 39, 40].

The decreasing eradication rate and increasing resistance to antibiotics support the importance of antimicrobial susceptibility testing and the need for new efficacious eradication regimens. Given that co-infecting *H. pylori* strains from a single patient frequently have different antibiotic resistance patterns, infection by multiple *H. pylori* strains may make it more difficult to interpret the results of antibiotic susceptibility tests and select appropriate eradication regimens. It has been suggested that heteroresistance of *H. pylori* in a single patient can contribute to treatment failure by conventional therapeutic regimens [10]. This implies that the presence of multiple infections should be considered as a possible cause of failure in the treatment of *H. pylori* infection. Besides, there are previous reports that show *H. pylori* isolates from different gastric sites in a single host presented different profiles of antibiotic resistance even though the isolates had similar or same genotype [41, 42]. This means that antibiotic resistance of *H. pylori* can also develop from pre-existing susceptible strains in a single host with topical variations. Therefore, it should be remembered that the etiology of heteroresistant antibacterial phenotypes is complex, and not only confined to infection with multiple strains.

Considering these points, it might be a reasonable strategy to obtain specimen from different gastric sites for *H. pylori* culture at the discretion of the clinician in certain clinical situations. These situations may include when multiple infection rate is not negligible and high antibiotic resistance is anticipated, for example, in areas with both high *H. pylori* prevalence and high antibiotic resistance. This strategy may also be useful when rescue therapy is considered.

There are some limitations in this study. First, only one type of primer was used. Although *H. pylori* strains can be distinguished by RAPD fingerprinting using a single primer, some recent studies have used several primers in combination to more accurately distinguish between different strains [10, 26]. However, antimicrobial susceptibility testing and the analysis of virulence factors supported the robustness of our RAPD fingerprinting results in identifying different *H. pylori* strains in this study.

Second, clinical records of subsequent eradication therapy and results in the patients were not available. Third, as the number of patients included was small, and the patients with single *H. pylori* infection were not included in the analysis, the results should be carefully interpreted. According to the enrollment criteria in our study, we only included patients with successful *H. pylori* isolation from both antrum and body. So this study does not give information comparing the clinical characteristics of *H. pylori* from patients with single infection and those with multiple infections. As the cases eventually showing only one positive culture in either antrum or body were not enrolled in this study, the true prevalence of multiple infection among the whole population with *H. pylori* infection may thus be lower.

## Conclusions

In conclusion, the presence of two *H. pylori* strains in a single patient was confirmed by RAPD fingerprinting. In the majority of cases, two *H. pylori* strains from a single host showed different virulence factors and antibiotic resistance patterns. Co-infection by multiple strains of *H. pylori* can increase the failure rate of eradication therapy and should be considered when interpreting the results of antimicrobial susceptibility tests.

## Additional files

Additional file 1: A list of primer sets. Contains primer sets used for genotyping *H. pylori* by PCR. (DOCX 21 kb)

Additional file 2: Primary data for virulence factor analysis. Contains agarose gel electrophoresis of the PCR-based *vacA* subtype, *iceA*, and *dupA* genotyping, amino acid sequences analysis of the *cagA* and *oipA* gene. (DOCX 1342 kb)

Additional file 3: Additional mechanistic study for antibiotic resistance. Contains mechanistic analysis of antibiotics resistance for clarithromycin and quinolone. (DOCX 17 kb)

## Data Availability

The datasets used and/or analysed during the current study are available from the corresponding author on reasonable request.

## Abbreviations

MIC: Minimum inhibitory concentration
PCR: Polymerase chain reaction
RAPD: Random amplified polymorphic DNA
