# Supplementary material for: The analysis of virulence factors and antibiotic resistance between Helicobacter pylori strains isolated from gastric antrum and body
Source: BMC Gastroenterol. 2019 Aug 7;19:140. doi: 10.1186/s12876-019-1062-5 (PMC6686454; doi:10.1186/s12876-019-1062-5)
Supplement: Supplementary file 1 — A list of primer sets. Contains primer sets used for genotyping H. pylori by PCR. (DOCX 21 kb) [file 12876_2019_1062_MOESM1_ESM.docx]

**Additional data 1**

| **Table S1.** Primer sets used for genotyping *H. pylori* by PCR | | | | |
| --- | --- | --- | --- | --- |
| Gene and region Amplified | Primer | Primer sequence (5ʹ→3ʹ) | Size (bp) of  PCR product | Reference |
| *ureA* | HP64-F | TCA CCC CAA AAG AGT TAG AC | 428 | ^[15]^ |
|  | HP64-R | GAA GTG TGA ACC GAT TGG AA |  |  |
| RAPD fingerprinting | 1281 | AAC GCG CAA C | - | ^[11]^ |
| *cagA* gene | F1 | GAT AAC AGG CAA GCT TTT GAG G | 349 | ^[17]^ |
|  | B1 | CTG CAA AAG ATT GTT TGG CAG A |  |  |
| CagA EPIYA | cagA2530S | GTT AAR AAT RGT GTR AAY GG | ^†^ | ^[16]^ |
|  | cagA3000AS | TTT AGC TTC TGA TAC CGC |  |  |
| *vacA* s1/s2 | VA1-F^‡^ | ATG GAA ATA CAA CAA ACA CAC | 259 (s1) | ^[17]^ |
|  | VA1-R^‡^ | CTG CTT GAA TGC GCC AAA C | 286 (s2) | ^[17]^ |
| *vacA* s1a | SAI-F^§^ | TCT YGC TTT AGT AGG AGC | 212 | ^[18]^ |
| *vacA* s1b | SS3-F^§^ | AGC GCC ATA CCC CAA GAG | 187 | ^[17]^ |
| *vacA* s1c | SIC-F^§^ | CTY GCT TTA GTR GGG YTA | 213 | ^[18]^ |
| *vacA* s2 | SS2-F | GCT AAC ACG CCA AAT GAT CC | 199 | ^[17]^ |
|  | VA1-R | CTG CTT GAA TGC GCC AAA C |  |  |
| *vacA* m1 | VAG-F | CAA TCT GTC CAA TCA AGC GAG | 570 | ^[19]^ |
|  | VAG-R | GCG TCT AAA TAA TTC CAA GG |  |  |
| *vacA* m2 | VAG-F | CAA TCT GTC CAA TCA AGC GAG | 645 | ^[19]^ |
|  | VAG-R | GCG TCT AAA TAA TTC CAA GG |  |  |
| *vacA* i1 | VacF1 | GTT GGG ATT GGG GGA ATG CCG | 432 | ^[20]^ |
|  | C1R | TTA ATT TAA CGC TGT TTG AAG |  |  |
| *vacA* i2 | VacF1 | GTT GGG ATT GGG GGA ATG CCG | 426 | ^[20]^ |
|  | C2R | GAT CAA CGC TCT GAT TTG A |  |  |
| *oipA* | HP0638-F | GTT TTT GAT GCA TGG GAT TT | 401 | ^[21]^ |
|  | HP0638-R | GTG CAT CTC TTA TGG CTT T |  |  |
| *dupA* | A-F | ATA GCG ATA ACC AAC AAG AT | 662 | ^[22]^ |
|  | A-R | AAG CTG AAG CGT TTG TAA CG |  |  |
|  | B-F | ATT CAC GCC TAA GAC CTC A | 1115 | ^[22]^ |
|  | B-R | AAG CTG AAG CGT TTG TAA CG |  |  |
| *iceA1* | iceA1-F | GTG TTT TTA ACC AAA GTA TC | 247 | ^[14]^ |
|  | iceA1-R | CTA TAG CCA STY TCT TTG CA |  |  |
| *iceA2* | iceA2-F | GTT GGG TAT ATC ACA ATT TAT | 229 or 334 | ^[14]^ |
|  | iceA2-R | TTR CCC TAT TTT CTA GTA GGT |  |  |
| 23S rRNA | 23S-F  23S-R | AGC GAT GTG GTC TCA GCA  CAA GGG TGG TAT CTC AAG G | 415 | ^[6]^ |
| *gyrA* | gyrA-F  gyrA-R | TTT AGC TTA TTC AAT GAG CGT  GCA GAC GGC TTG GTA GAA TA | 428 | ^[6]^ |
| *gyrB* | gyrB-F  gyrB-R | GCA AAA GCC AGA GAA GCC A  ACA TGC CCT TGT TCA ATC AGC | 444 | ^[6]^ |
| Y is C or T, M is A or C, S is C or G, and R is A or G. ^†^ Positions 582453–582977 with reference to the *H. pylori* 26695 genome. ^‡^ The product amplified using this primer pair was divided into s1 and s2 according to the molecular size. ^§^ Used with primer VA1-R. | | | | |
