# Supplementary material for: The analysis of virulence factors and antibiotic resistance between Helicobacter pylori strains isolated from gastric antrum and body
Source: BMC Gastroenterol. 2019 Aug 7;19:140. doi: 10.1186/s12876-019-1062-5 (PMC6686454; doi:10.1186/s12876-019-1062-5)
Supplement: Supplementary file 2 — Primary data for virulence factor analysis. Contains agarose gel electrophoresis of the PCR-based vacA subtype, iceA, and dupA genotyping, amino acid sequences analysis of the cagA and oipA gene. (DOCX 1342 kb) [file 12876_2019_1062_MOESM2_ESM.docx]

**Additional data 2:** Primary data for virulence factor analysis

**Supplementary Figures**

**Figure S1.** Agarose gel electrophoresis of the PCR-based *vacA* subtype genotyping. M, 100 bp DNA ladder (Elpisbio); Numbers 1–10 indicate individual patients; A, stomach antrum; B, stomach body. **A.** *vacA s1* gene (259 bp). All strains were positive. **B.** *vacA s1a* gene (212 bp). 1B, 2B, 3A, 3B, 5B and 8B are *vacA s1a* positive while remaining strains are negative for this gene. **C.** *vacA s1c* gene (213 bp). 1A, 2A, 4A, 4B, 5A, 6A, 6B, 7A, 7B, 8A, 9A, 9B, 10A and 10B 8B are *vacA s1c* positive while remaining strains are negative for this gene. **D.** *vacA m1* (570 bp) and *m2* (645 bp) gene. 5A, 8B, 9A and 9B are *vacA m2* positive while remaining strains are *vacA m1* positive. **E.** *vacA i1* gene (432 bp). 5A and 8B are *vacA i1* gene negative while remaining strains are positive for this gene. **F.** *vacA i2* gene (426 bp). 5A and 8B are *vacA i2* gene positive while remaining strains are negative for this gene.

**Figure S2.** Amino acid sequences analysis of the *cagA* gene. Numbers 1–10 indicate individual patients; A, stomach antrum; B, stomach body. 5A, 8A, 8B, 10B strains, respectively, show *cagA* with EPIYA-ABC pattern while remaining strains are EPIYA-ABD. The different EPIYA patterns in the same patient were present in 5 (5A, 5B strains) and 10 (10A, 10B strains).

**Figure S3.** Agarose gel electrophoresis of the PCR-based *iceA* and *dupA* genotyping. M, 100 bp DNA ladder (Elpisbio); Numbers 1–10 indicate individual patients; A, stomach antrum; B, stomach body. **A.** *iceA1* gene (247 bp). All strains were positive. **B.** *dupA* gene (dupA-A primer, 401 bp). **C.** *dupA* gene (dupA-B primer, 1115 bp). 5B, 8A, 8B and 10A were *dupA* positive while remaining strains were negative for this gene.

**Supplementary Tables**

**Table S2.** Nucleotide and deduced amino acid sequences of the signal peptide-coding region of the *oipA* gene.

**Figure S1.**


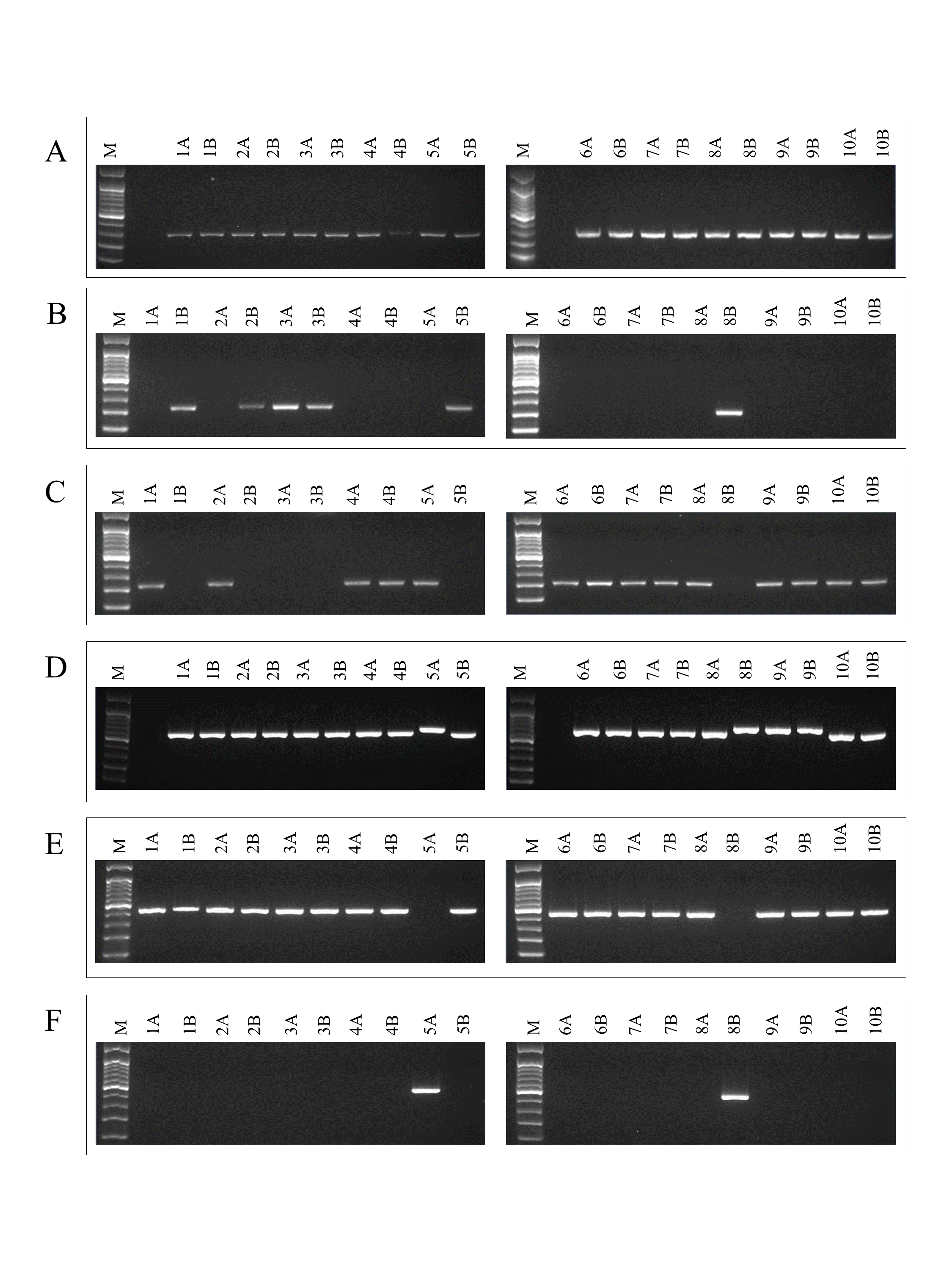


**Figure S2.**


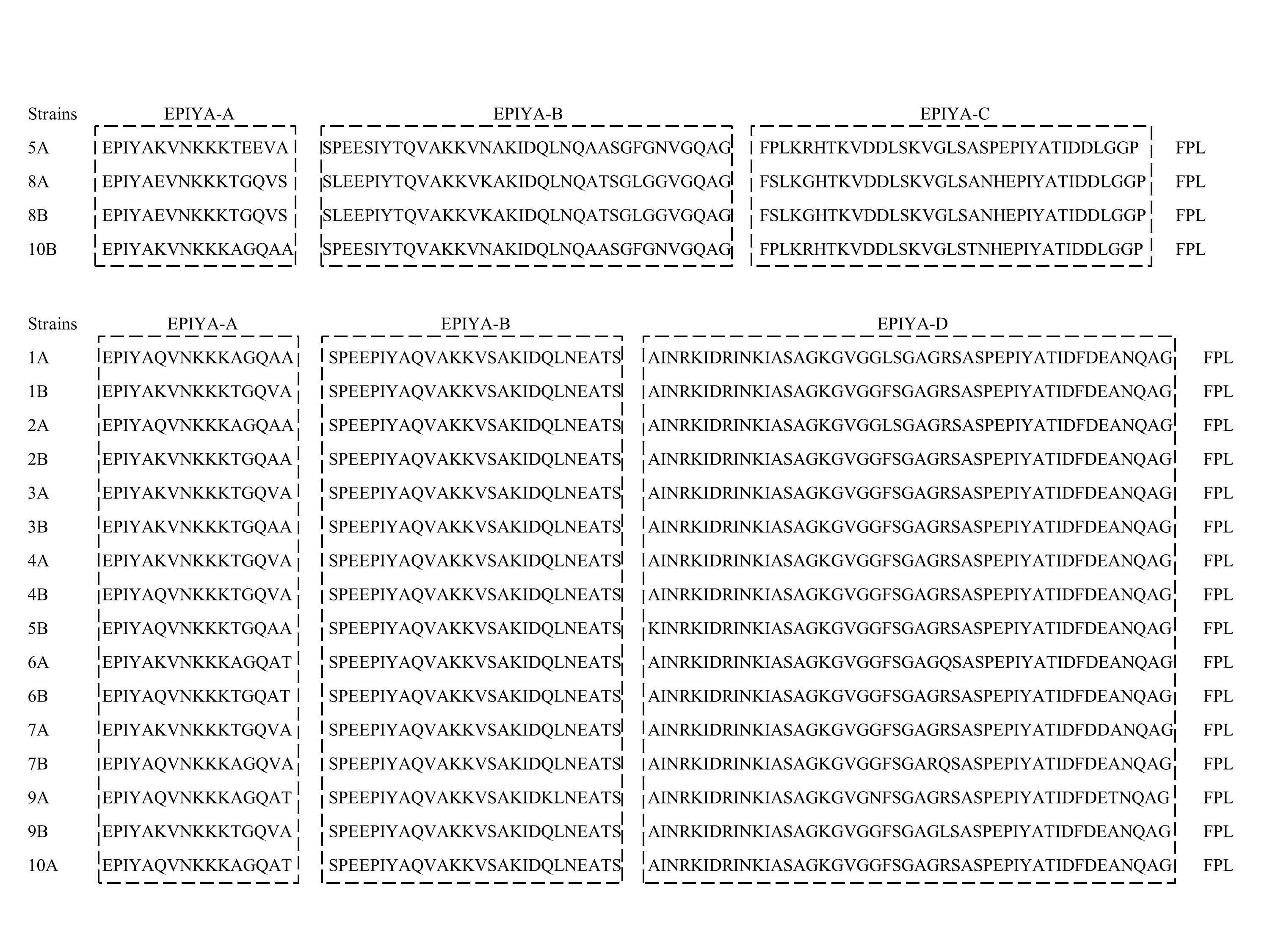


**Figure S3.**


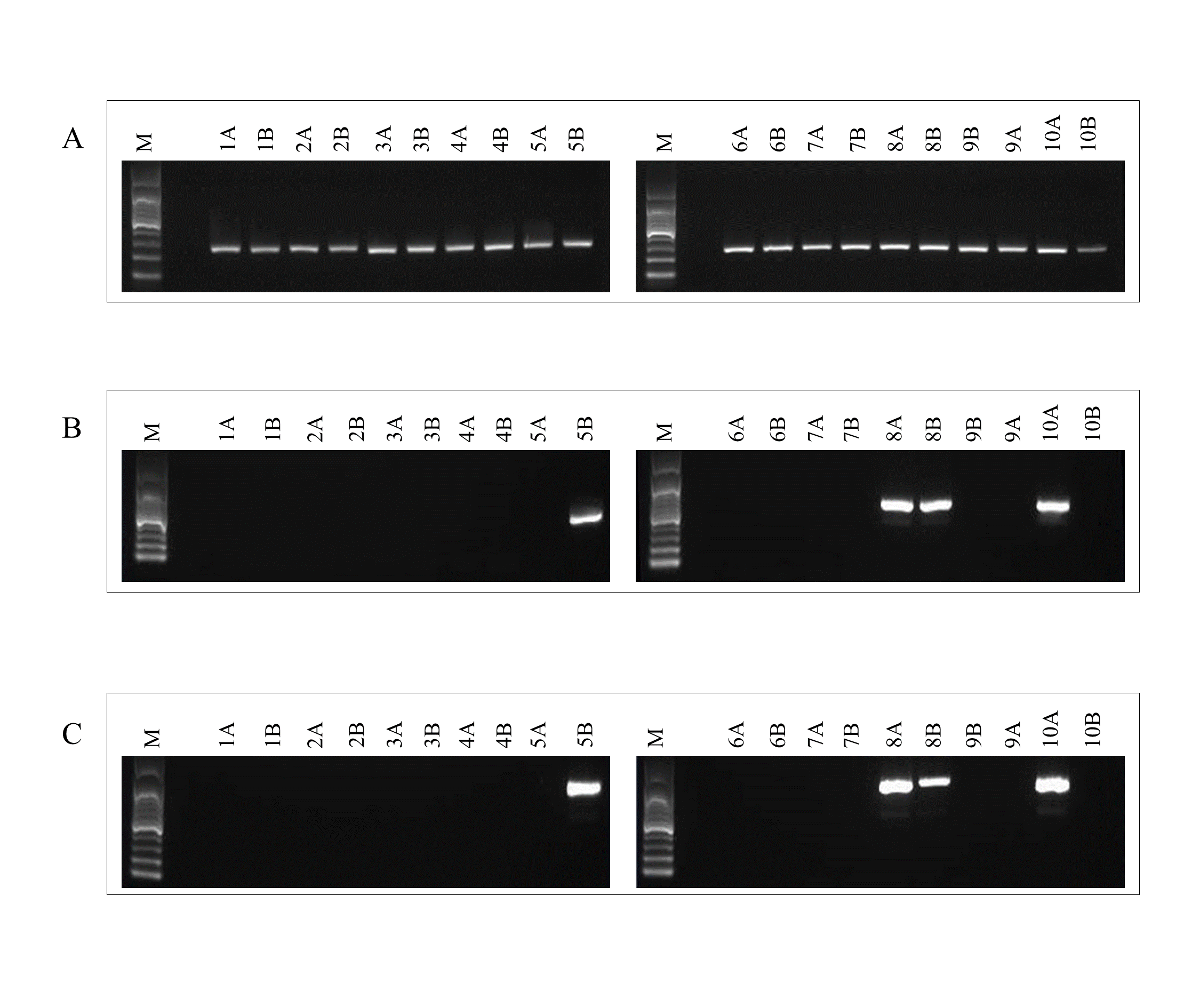


| **Table S2.** Nucleotide and deduced amino acid sequences of the signal peptide-coding region of the *oipA* gene. | | |  |
| --- | --- | --- | --- |
| Strains | Sequence of signal peptide encoding region of *oipA* | Number of CT repeats | Gene status |
| 1A | ATGAAAAAAACCCTTTTACTAACTCTCTTTTTCTCGTTTTGGCTC | 3+1 | on |
|  | M K K T L L L T L F F S F W L |  |  |
| 1B | ATGAAAAAAGCCCTTTTACTAACTCTCTTTTTCTCGTTTTGGCTC | 3+1 | on |
|  | M K K A L L L T L F F S F W L |  |  |
| 2A | ATGAAAAAAACCCTTTTACTAACTCTCTTTTTCTCGTTTTGGCTC | 3+1 | on |
|  | M K K T L L L T L F F S F W L |  |  |
| 2B | ATGAAAAAAGCTCTCTTACTAACTCTCTTTTTCTCGTTTTGGCTC | 3+1 | on |
|  | M K K A L L L T L F F S F W L |  |  |
| 3A | ATGAAAAAAGCCCTTTTACTAACTCTCTTTTTCTCGTTTTGGCTC | 3+1 | on |
|  | M K K A L L L T L F F S F W L |  |  |
| 3B | ATGAAAAAAGCTCTCTTACTAACTCTCTTTTTCTCGTTTTGGCTC | 3+1 | on |
|  | M K K A L L L T L F F S F W L |  |  |
| 4A | ATGAAAAAAACCCTTTTACTCTTTCTGTCTTTCTCGTTTTGGCTC | 2+1+1+1 | on |
|  | M K K T L L L F L S F S F W L |  |  |
| 4B | ATGAAAAAAACCCTTTTACTAACTCTCTTTTTCTCGTTTTGGCTC | 3+1 | on |
|  | M K K T L L L T L F F S F W L |  |  |
| 5A | ATGAAAAAAACCCTTTTACTAACTCTCTTTTTCTCGTTTTGGCTC | 3+1 | on |
|  | M K K T L L L T L F F S F W L |  |  |
| 5B | ATGAAAAAAACCCTTTTACTAACTCTCTTTTTCTCGTTTTGGCTC | 3+1 | on |
|  | M K K T L L L T L F F S F W L |  |  |
| 6A | ATGAAAAAAACCCTTTTACTCTTTCTGTCTCTCTCGTTTTGGCTC | 2+1+3 | on |
|  | M K K T L L L F L S L S F W L |  |  |
| 6B | ATGAAAAAAACCCTTTTACTAACCCTCTTTTTCTCGTTTTGGCTC | 2+1 | on |
|  | M K K T L L L T L F F S F W L |  |  |
| 7A | ATGAAAAAAACCCTTTTACTAACTCTCTTTTTCTCGTTTTGGCTC | 3+1 | on |
|  | M K K T L L L T L F F S F W L |  |  |
| 7B | ATGAAAAAAACCCTTTTACTAACTCTCTTTTTCTTGTTTTGGCTC | 3+1 | on |
|  | M K K T L L L T L F F L F W L |  |  |
| 8A | ATGAAAAAAGCTCTCTTACTAACTCTCTCTCTCCCTCTCTCGTTTTGGCTC | 5+3 | on |
|  | M K K A L L L T L S L P L S F W L |  |  |
| 8B | ATGAAAAAAGCTCTCTTACTAACTCTCTCTCTCCCTCTCTCGTTTTGGCTC | 5+3 | on |
|  | M K K A L L L T L S L P L S F W L |  |  |
| 9A | ATGAAAAAAACCCTTTTACTCTTTCTGTCTTTCTCGTTTTGGCTC | 2+1+1+1 | on |
|  | M K K T L L L F L S F S F W L |  |  |
| 9B | ATGAAAAAAACCCTTTTACTCTTTCTCTCTTTCTTGTTTTGGCTC | 2+3+1 | on |
|  | M K K T L L L F L S F L F W L |  |  |
| 10A | ATGAAAAAAACCCTTTTACTCTTTCTCTTTTTCTCGTTTTGGCTC | 2+2+1 | on |
|  | M K K T L L L F L F F S F W L |  |  |
| 10B | ATGAAAAAAGCTCTCTTACTAACTCTCTTTTTCTCGTTTTGGCTC | 3+1 | on |
|  | M K K A L L L T L F F S F W L |  |  |

Numbers 1–10 indicate individual patients; A, stomach antrum; B, stomach body; Number of CT repeats, cytosine-thymine repeated in the nucleotide sequence; on, functional status
