# Supplementary material for: The analysis of virulence factors and antibiotic resistance between Helicobacter pylori strains isolated from gastric antrum and body
Source: BMC Gastroenterol. 2019 Aug 7;19:140. doi: 10.1186/s12876-019-1062-5 (PMC6686454; doi:10.1186/s12876-019-1062-5)
Supplement: Supplementary file 3 — Additional mechanistic study for antibiotic resistance. Contains mechanistic analysis of antibiotics resistance for clarithromycin and quinolone. (DOCX 17 kb) [file 12876_2019_1062_MOESM3_ESM.docx]

**Additional data 3**

| **Table S3.** Mechanistic analysis of antibiotics resistance for clarithromycin and quinolone. | | | | | | |
| --- | --- | --- | --- | --- | --- | --- |
| Strain no. | V domain of 23S rRNA gene mutation | *gyrA* mutation | *gyrB* mutation |  |  |  |
| 1A | X | T261A | X |  |  |  |
| 1B | X | X | X |  |  |  |
| 2A | X | T261A | X |  |  |  |
| 2B | X | X | X |  |  |  |
| 3A | X | X | X |  |  |  |
| 3B | X | X | X |  |  |  |
| 4A | X | X | X |  |  |  |
| 4B | X | X | X |  |  |  |
| 5A | X | X | X |  |  |  |
| 5B | X | X | X |  |  |  |
| 6A | X | X | X |  |  |  |
| 6B | X | X | X |  |  |  |
| 7A | X | X | X |  |  |  |
| 7B | X | X | X |  |  |  |
| 8A | A2142G | G271A | X |  |  |  |
| 8B | A2142G | X | X |  |  |  |
| 9A | X | X | X |  |  |  |
| 9B | X | X | X |  |  |  |
| 10A | X | X | X |  |  |  |
| 10B | A2142G | X | X |  |  |  |
| The V domain of 23S rRNA, *gyrA* and *gyrB* gene sequence was analyzed to clarify the mechanism of antibiotic resistance (clarithromycin, quinolone). Numbers 1–10 indicate individual patients; A, stomach antrum; B, stomach body. | | | | | | |
